# Is DNA Methylation a Ray of Sunshine in Predicting Meningioma Prognosis?

**DOI:** 10.3389/fonc.2020.01323

**Published:** 2020-09-04

**Authors:** Lu Shen, Danfeng Lin, Lu Cheng, Sheng Tu, Haijian Wu, Weilin Xu, Yuanbo Pan, Xiaochen Wang, Jianmin Zhang, Anwen Shao

**Affiliations:** ^1^Department of Surgical Oncology, The Second Affiliated Hospital, Zhejiang University School of Medicine, Hangzhou, China; ^2^Department of Pathology, The Second Affiliated Hospital, Zhejiang University School of Medicine, Hangzhou, China; ^3^State Key Laboratory for Diagnosis and Treatment of Infectious Diseases, Collaborative Innovation Center for Diagnosis and Treatment of Infectious Diseases, The First Affiliated Hospital, College of Medicine, Zhejiang University, Hangzhou, China; ^4^Department of Neurosurgery, The Second Affiliated Hospital, Zhejiang University School of Medicine, Hangzhou, China; ^5^Department of Breast Surgery, Zhejiang Provincial People’s Hospital, People’s Hospital of Hangzhou Medical College, Hangzhou, China; ^6^Brain Research Institute, Zhejiang University, Hangzhou, China; ^7^Collaborative Innovation Center for Brain Science, Zhejiang University, Hangzhou, China

**Keywords:** meningioma, methylation, classification, prognosis, gene

## Abstract

Meningioma is the most common intracranial tumor, and recent studies have drawn attention to the importance of further research on malignant meningioma. According to the World Health Organization (WHO) grading, meningioma is classified into 15 subtypes with three grades of malignancy. However, due to a lack of descriptions of molecular subtypes, genetic mutations, or other features, there were deficiencies in the WHO classification. The DNA methylation-based meningioma classification published in 2017 used DNA copy number analysis, mutation profiling, and RNA sequencing to distinguish six clinically relevant methylation classes, which contributed to a better prediction of tumor recurrence and prognosis. Further studies indicated that gene variation and gene mutations, such as those in neurofibromin 2 *(NF2)* and *BRCA1*, were related to the high WHO grade, malignant invasion, and recurrence. Among the mutant genes described above, some have been associated with differential DNA methylation. Herein, we searched for articles published in PubMed and Web of Science from January 2000 to May 2020 by entering the keywords “meningioma,” “methylation,” and “gene mutation,” and found a number of published studies that analyzed DNA methylation in meningiomas. In this review, we summarize the key findings of recent studies on methylation status and genetic mutations of meningioma and discuss the current deficits of the WHO grading. We also propose that a methylation-based meningioma classification could provide clues in the assessment of individual risk of meningioma recurrence, which is associated with clinical benefits for patients.

## Introduction

Meningiomas, tumors of the meningeal coverings of the brain and spinal cord, are the most common intracranial tumors. According to the World Health Organization (WHO) classification, 80% of meningiomas are grade I and are considered benign. The remaining 20% are grade II and III with a malignant histological tendency (1). Although the WHO grade is considered to be the most reliable indicator in predicting meningioma prognosis (2, 3), there is significant variation with regards to the risk of recurrence for individual patients (4, 5).

Recently, many studies have demonstrated that epigenetic changes, especially DNA methylation, as well as genetic mutations are related to tumor prognosis (4) (Table 1). A totally new classification based on DNA copy number analysis, mutational profiling, and RNA sequencing has been used to distinguish six individual clinically relevant methylation classes (6) to better predict tumor recurrence and prognosis. A series of studies have identified methylation profiling as a marker of malignancy or poor survival rates and genetic mutations as an indicator of histology grade. However, there has been no significant evidence illustrating a relationship between DNA methylation and genetic mutation. The most recent papers suggest that many potential genes, like *NDRG2* and *MAL2*, are related to DNA methylation, but whether they can predict prognosis remains controversial.

**TABLE 1:** Key findings on meningiomas in the last two decades.

Year	Number of meningiomas sample					Key findings	References
	Total	Grade I	Grade II	Grade III	Comment		
2004	72	/	/	/	58 benign, 10 atypical and 4 anaplastic meningiomas	The relationship between p53 gene mutation and p14 (ARF) gene methylation	(6)
	50	20	14	16	/	The methylation of p16 (INK4a)	(7)
	60	33	24	3	/	The methylation of TP73	(8)
2005	48	/	/	/	16 benign, 19 atypical, and 13 anaplastic meningiomas	The promoter hypermethylation is associated with atypical and anaplastic meningiomas; the methylation of MGMT	(9)
	44	15	11	8	10 meningiomas (grade I and grade II)	Hypermethylation of the NDRG2 promoter	(10)
2006	25	/	/	/	Meningiomas without NF2 involvement	The methylation of NF2, p14 (ARF), CDH1, BRCA1, RB1	(11)
2007	40	22	11	7	/	The methylation status of p73 or RASSF1A along with 1p LOH may result in the malignant transformation of a meningioma	(12)
2009	26	10	7	9	209 tumors from 13 other tumor types; two human malignant meningioma cell lines: IOMM-Lee21 and KT21-MG1	Aberrant methylation of the CpG island of WNK2 was associated with decreased expression in primary tumors	(13)
2010	50	27	11	12	/	The methylation of TIMP3	(14)
2011	65	26	27	12	/	uPA Promoter Methylation	(15)
2012	131	100	28	3	/	The methylation of HOXA 7, 9, and 10 were associated with histopathology and clinical aggressiveness parameters	(16)
	36	16	17	3	/	The methylation of MGMT, CDKN2A, GSTP1, and THBS1	(17)
	50	20	16	14	/	Hypermethylation of the promoter of hMLH1 is associated with the tumor grade	(18)
2013	33	30	2	1	Discovery set	The methylation of IGF2BP1 and PDCD1	(19)
	12	6	5	1	Verification set		
	19	10	5	4	/	Global DNA hypomethylation and the MAL2 gene	(20)
2015	44	33	2	9	/	The methylation of CDKN2B, RASSF1A, RUNX3, AND GATA6	(21)
2016	/	/	/	/	*de novo* tumors: 41 meningiomas and 33 hemangiopericytomas; recurrent tumors: 37 meningiomas and 5 hemangiopericytomas	The promoter methylation of hTERT was positively correlated with WHO grade and hTERT expression	(22)
2017	497	/	/	/	309 samples of other extra-axial skull tumors	DNA Methylation-Based Classification	(23)
	89	54	34	1	Test group	Robust DNA methylation signatures in meningioma were correlate with CNAs and could stratify patients by recurrence risk	(24)
	51	36	9	6	Validation group		
2019	282	/	/	/	Training cohort: 228; the first validation cohort: 54	Established a 5-year meningioma recurrence score	(4)

## Methods

We reviewed the relevant literature on PubMed and Web of Science that had been published between January 2000 and May 2020. We identified 599 studies addressing aberrant DNA methylation and genetic mutation in meningiomas. The words we searched included “meningioma,” “methylation,” and “gene mutation.” After analyzing all relevant articles, we found genes-of-interest and searched those genes on PubMed and Web of Science to acquire pertinent information.

## The Genetic Mutations in WHO Grade

In recent years, several aberrant gene mutations have been reported in meningiomas. The new WHO grade (2016 version) adds this molecular feature to its criteria and tries to give a clear description of different histological subtypes (1). Although the WHO grade defines characteristics of each subtype, the deficiencies of this classification have gradually been revealed.

### The WHO Grade – Subtypes and Molecular Features

To better predict the prognosis of meningiomas, the WHO grade 2016 criteria, which emphasized mitotic activity and brain invasion, classified meningiomas into three pathological grades with 15 subtypes (Table 2). These included grade I tumors of nine subtypes (meningothelial, fibrous, transitional, psammomatous, angiomatous, microcystic, secretory, lymphoplasmacytic-rich, and metaplastic subtypes), grade II of three histological subtypes (chordoid, clear cell, and atypical), and grade III meningiomas of three subtypes (papillary, rhabdoid, and anaplastic) (7). Each grade has unique molecular features that have been reviewed by previous studies (1, 8). Grade I meningiomas show high mutation rates of some genes (Table 2), including neurofibromin 2 (*NF2*), the proto-oncogene v-Akt murine thymoma viral oncogene homolog 1 (*AKT1*), the ubiquitin ligase tumor necrosis factor receptor-associated factor 7 *(TRAF7*), the oncogene phosphatidylinositol-4, 5-bisphosphate 3-kinase catalytic subunit alpha (*PIK3CA*), the pluripotency transcription factor Kruppel-like factor 4 (*KLF4*), and the gene for the catalytic subunit of RNA polymerase II (*POLR2A*) (9, 10). *TRAF7* mutations occur in 25% of WHO grade I and II meningiomas (11). Other genetic mutations have also been reported, including phosphatase and tensin homolog (*PTEN*), cyclin-dependent kinase inhibitor 2A (*CDKN2A*), and cyclin-dependent kinase inhibitor 2B (*CDKN2B*) genes which can be found in grade III meningiomas (12). High-grade meningiomas are characterized by more mutations than grade I meningiomas (9).

**TABLE 2:** The WHO grade (2016 criteria) (8).

WHO grade (2016 criteria)	Histological subtype	Gene mutations
I	Meningothelial	TRAF7, AKT1, POLR2A, PIK3CA
	Fibrous (fibroblastic)	NF2
	Transitional (mixed)	NF2, AKT1, PIK3CA
	Psammomatous	NF2
	Angiomatous	–
	Microcystic	–
	Secretory	KLF4, TRAF7
	Lymphoplasmacyte-rich	–
	Metaplastic	–
II	Chordoid	–
	Clear cell	SMARCE1
	Atypical	NF2, TRAF7, AKT1
III	Papillary	–
	Rhabdoid	BAP1
	Anaplastic (malignant)	NF2

Different histological subtypes of meningiomas also harbor various characteristics. For example, secretory meningiomas show frequent co-mutations of the *KLF4* and *TRAF7* genes (13, 14), while clear cell meningiomas show SWI/SNF-related, matrix-associated, actin-dependent regulators of chromatin, subfamily e, member 1 (*SMARCE1*) mutations (15). A subset of rhabdoid meningiomas reveals poor outcomes by inactivating the *BAP1* gene, compared to patients with *BAP1*-negative rhabdoid meningiomas (16). Therefore, adding genetic mutations into the WHO classification system allows each subtype of meningiomas to be more precisely characterized.

Regarding prognosis, histologic grade (the WHO grade) and the extent of surgical resection (the Simpson grade) were considered the two most important prognostic variables (3, 17). Heald et al. showed that gross-total resection significantly decreases the risk of recurrence (2), suggesting the importance of the Simpson grade in meningioma recurrence. Studies have also demonstrated that, with the exception of other external conditions (i.e., therapeutic regimens), a combination of the WHO grade and the Simpson grade has better clinical value to predict the prognosis of different grades of meningiomas. Grade I meningiomas have a 10-year overall survival rate of 80% and a progression-free survival rate (PFS) of approximately 74 to 96% (18–20). Grade II meningiomas have an easier tendency for recurrence, and their 10-year overall survival (OS) and PFS are between 53–79% and 23–78%, respectively. Comparatively, grade III meningiomas are more aggressive and significantly associated with brain invasion, with a 10-year OS of 14 to 34% and PFS of 0%, even for those that undergo gross-total resection (3, 5). These results show that a higher histologic grade is associated with a poorer survival rate, and for patients with a WHO grade III meningiomas, subtotal resection indicates a worse prognosis. Clinicians have utilized these methods to predict the prognosis of patients. However, some deficits in the WHO grading system remain.

### The Deficiencies of WHO Grade

Usually, the risk of recurrence is predicted based on the WHO grade. However, as many malignant tumors are underestimated in clinical practice, the actual risk of recurrence is higher than the predicted risk. Thus, a new grading system is needed. Based on clinical experience, meningiomas of the same grade can exhibit totally different biologic behaviors. For example, some meningiomas that are designated as benign can recur within a short amount of time, while other meningiomas with high-grade features may hardly ever exhibit recurrence (21, 22). It has also been reported that some grade I meningiomas exhibit early or frequent recurrence and metastasis to distant organs, such as the lungs (23–26). As the prediction of outcomes through the use of the WHO and Simpson classifications to predict the prognosis of meningiomas has been inconsistent with overall results, there appear to be other factors affecting the progression of meningiomas.

### Observations of Epigenetic Alteration and Gene Methylation in Meningiomas

Indeed, there is an urgent need for a more accurate subclassification covering histological and surgical resection assessments that reflects the potential malignant characteristics of meningiomas. Recently, it has become a trend to study the molecular features of different meningioma subtypes to find out potential biomarkers that suggest worse progression. Chromosomal structural variation and genetic mutations have become a research hotspot. Mawrin et al. found few chromosomal alterations in WHO grade I tumors, but frequent alterations of karyotype and copy number in WHO grade II tumors. Losses of chromosomes 1p, 6q, 9p, 10, 14q, and 18q, and gains of chromosomes 1q, 9q, 12q, 15q, 17q, and 20q have been found across grade III meningiomas (27). Different genes contribute to different meningiomas’ subtypes. These genes include *TRAF7*, *AKT1*, *POLR2A*, *PIK3CA*, *SMO*, *KLF4*, *SMARCB1*, *BAP1*, and *NF2* (11, 16, 28–32). Each subtype of meningiomas has its own molecular features, but some genetic mutations (including *NF2*, *TRAF7*, *AKT1*, and *PIK3CA*) can be found among several subtypes of meningiomas, which confuse the judgment of tumor evolution.

Additionally, epigenetic alteration has become increasingly important in tumor occurrence and evolution. These alterations appear in meningiomas without causing the aforementioned gene mutations (33–35). Epigenetic changes caused by complicated mechanisms modulate heritable gene expression without altering the primary sequence of DNA (36). These changes include DNA methylation, microRNA interactions, histone packaging, and chromatin restructuring. Among these, abnormal DNA methylation is a chemical modification process mediated by DNA methyltransferase (DNMT). It can cause gene silencing and a decline in expression by blocking the transcriptional machinery from accessing the DNA (37, 38). As aberrant DNA methylation often occurs in the early stage of tumorigenesis, it can be detected in the early stage of disease (37, 39). The methods currently used to detect DNA methylation include bisulfite methods [such as whole-genome bisulfite sequencing (WGBS), reduced representation bisulfite sequencing (RRBS)], and Illumina EPIC methylation array profiling) and non-bisulfite methods (40). According to research data, global detection of abnormal methylation genes can provide biomarkers of clinical potential for the prognosis of certain cancers, such as lung cancer and colorectal cancer (41, 42). Actually, DNA methylation is a common event in meningiomas, as approximately 77% harbor at least one differentially methylated gene and 25% experience alterations of three or more genes (33). Although gene methylation can be detected across all grades of meningiomas, the frequency varies between benign and malignant tumors, and genes (e.g., *TIMP3*, *GSTP1*, *MEG3*, *HOXA6*, *HOXA9*, *PENK*, *WNK2*, and *UPK3A* genes) have an increased frequency according to the WHO grade (12). Several studies indicate that a large number of genes are methylated in meningiomas (43–46) and uncover the relationship between genes and WHO grade, which lays a solid foundation for the methylation classification of this tumor type.

## The Methylation Classes in Meningiomas

For meningiomas, a large proportion of previous studies (45–48) have focused on aberrantly methylated genes, but few have studied classification based on methylation. The methylation grading may not be being utilized effectively. In 2017, new methylation classes were published and were considered a more reliable classification system compared to the WHO’s grading. Some newly discovered genes were not included in this classification; subsequent supplementary experiments are in preparation.

### The Old Methylation Classes

Based on methylation profiling, one study subclassified WHO grade I and II meningiomas into three methylation clusters (39), and they did not include grade III meningiomas. Another study used robust methylation signatures (283-bMMC model) to distinguish two clinical–biological subgroups, including one clinically favorable prognostic subgroup (MM-FAV) and another clinically unfavorable meningioma methylation subgroup (MM-UNFAV) (Table 3). The Kaplan–Meier survival analysis showed that tumors in the MM-UNFAV group had significantly shorter recurrence times compared to MM-FAV. After adjusting for relevant morphological, clinical, and molecular variables, 283-bMMC subgroups did not show significance in predicting recurrence, but a subset (64-MMP) proved to be a meningioma methylation predictor (22). Both studies provide innovative classifications of risk-related meningiomas, suggesting a proof-of-concept that DNA methylation profiles act as an important prognostic marker in meningiomas (33, 49).

**TABLE 3:** The 283-bMMC model subgroups (2017).

Basic Methylation Classifier (bMMC)	Subgroups	CNA Patterns	Median RFS (in validation groups)
283-bMMC	MM-FAV	+1p, −22q	16.35 years
	MM-UNFAV	−1p, +1q, −2p, −3p/+3, −4, +5, −6q, +9, −10, +12, +13q, −14q, +15q, −16, −18, +20, +21q, +22q	8.27 years

### The New Methylation Classes

Sahm et al. established a totally new classification on meningiomas, characteristics of which included DNA copy number analysis, mutational profiling, and RNA sequencing. After studying 497 meningiomas and 309 samples of other extra-axial skull tumors, they found that using DNA methylation data clearly distinguishes meningiomas from other tumors, indicating the specificity of DNA methylation for meningiomas. Based on the molecular spectrum of meningiomas, 497 meningiomas were divided into two major groups:group A and B. Tumors in group A followed a mainly benign clinical course, while tumors in group B showed an intermediate to malignant clinical course. However, whether these two groups develop from distinct cells of origin needs to be confirmed.

In additional studies, researchers found four subgroups in group A and two subgroups in group B. The six methylation classes were designated as MC ben-1, MC ben-2, MC ben-3, MC int-A, MC int-B, and MC mal (Table 4). MC ben-1, MC ben-2, and MC ben-3 were benign tumors. MC int-A and MC int-B were intermediate tumors and had higher rates of progression and recurrence. MC mal was distinguished as a malignant tumor with a high possibility of progression and recurrence. Generally, the DNA-based classification is different from the WHO grade. However, researchers noted an enrichment of grade I tumors in MC ben-1, MC ben-2, and MC ben-3. When methylation subgroups were scattered among all WHO grades, the new classification covered more molecular features, such as DNA methylation profile. The new classification was likely to predict meningioma prognosis better, and its predicted Kaplan-Meier survival curves were more accurate than those predicted by the WHO grade (6). For example, in the 497 samples collected by Sahm et al., most WHO grade II tumors belonged to the MC int-A and MC int-B classes, while a portion of them belonged to the MC ben-1 methylation class. The survival time in the MC ben-1 methylation class was lower compared to the MC int-A and MC int-B classes. Analogously, most of the WHO grade III tumors belonged to the MC mal methylation class. However, a subset of WHO grade III tumors were also classified as MC int-B. For this tumor type, data demonstrated a significant difference between the PFS of two methylation classes, with MC mal tumors showing a worse PFS than MC int-B tumors. This conclusion was in accordance with that of Sievers et al., who used 28 chordoid meningiomas to illustrate that DNA methylation classification had higher accuracy in outcome prediction than the WHO grading (50).

**TABLE 4:** The methylation-based classification (2017) and associations with WHO grade.

Epigenetic groups	Methylation class	Subgroups	WHO grade
Group A	Benign	MC ben-1	Fibroblastic, Transitional, Atypical
		MC ben-2	Secretory, Transitional, Meningothelial
		MC ben-3	Angiomatous, Transitional, Atypical
	Intermediate	MC int-A	Fibroblastic, Transitional, Atypical
Group B		MC int-B	Atypical, Anaplastic
	Malignant	MC mal	Anaplastic

Compared to the WHO grade, applying methylation profiling for meningioma classification may have a higher value as it helps identify progressive tumors among low-grade meningiomas and stable tumors among high-grade meningiomas. It signifies a potential capability to reduce undertreatment or overtreatment. For instance, a patient with a WHO grade I meningioma may be treated by clinicians as a patient with benign tumors due to histological identification, but there is still a small chance of recurrence. This means the traditional WHO grading cannot distinguish the potential malicious tumors from stable tumors within the same grade. Therefore, malignant tumors that are considered as benign may be underrated (6). In conclusion, the new methylation classification can help clinicians choose optimal treatment regimens (including chemotherapy, radiotherapy, or molecular targeted therapy) for patients with the same grade and different methylation profiles.

Besides meningiomas, methylation classification has been proven to be more relevant than histological grading in other solid tumors, such as gliomas (1, 51), thus revealing the importance of methylation profiling in tumor classification. Although the new parameters for predicting prognosis exhibited advantages over other methods, it still has some limitations. First, the collected 497 meningiomas were retrospectively analyzed, making the results not so convincing. It is necessary to conduct a prospective study to put this new classification into practice and track the results, as well as learn the feasibility of the new classification. Second, insufficient clinical data and technical limitations have made the result not very reliable. In the study, only 228 samples’ clinical data were obtained, and only 303 samples have been sequenced to study the relationship between genetic mutations and methylation classes. Third, as this study does not include new genes discovered in recent years, such as *PPM1D* and *POLR2A*, tumors exhibiting these features were not classified into any subgroups.

Overall, the new classification system brings a new way to stratify, classify, and treat meningiomas. Although it is better than the previous grading system, more prospective studies that incorporate new genes are needed to improve its use clinically.

## Methylation Class and Histological Subtypes

Methylation classification based on the methylation profiles shows an improved predictive ability compared to the WHO grade and Simpson system. When discussing the relationship between these groups, the two independent systems overlap in some features. The newest studies have identified a lot of genes that could be used as biomarkers for meningiomas, and some of them may influence the progression via DNA methylation. Others may have distinctive mechanisms. The genes that are methylated in meningiomas have different functions on tumor progression. However, some of them, such as *MGMT*, need more research in order to prove their function on meningiomas.

### The Relationship Between Methylation Class and Histological Subtypes

There are two different patterns that characterize methylation class and histological subtypes (Table 4). First, methylation class is significantly associated with a subset of histological subtypes. MC int-A and MC int-B classes are mainly composed of atypical meningiomas (WHO grade II), while the remaining (23%) of atypical meningiomas belong to the benign class MC ben-1. Additionally, 76% of MC mal were anaplastic meningiomas (WHO grade III), but anaplastic meningiomas also exist in both MC int-B (47%) and MC int-A (12%). Second, methylation class samples widely exist among all corresponding variants, including the rhabdoid and papillary meningiomas (MC ben-3, MC int-B, and MC int-A), transitional meningiomas (MC int-A and MC int-B), fibroblastic meningiomas (MC ben-1), and meningothelial meningiomas (MC ben-2) (6). High-grade meningioma histology more frequently appears in higher methylation classes (MC int-A is higher than MC ben-2). The aforementioned two patterns show that the relationship between methylation classes and histological types is complex. Even when the features of genetic mutation in each methylation subgroups were studied, results were only able to roughly indicate which subgroup harbored which kind of aberrant genes or cytogenetics. Although Paramasivam et al. have studied mutation patterns in epigenetic subgroups (52), we cannot infer the inner connections due to the limited amount of studies. Hence, future studies should analyze large-samples with integrated data in each methylation class.

Nevertheless, this issue has remained controversial due to a study that reported that DNA methylation of a gene is not strongly correlated to gene expression during the malignant transformation of meningiomas (49). Nowadays, the hypothesis is accepted that both mechanisms can occur independently or co-exist in one sample, while various meningiomas harbor different situations. For example, WHO Grade I and II meningiomas present relatively more aberrantly methylated loci than genetically altered loci (53). As previously shown, aberrant promoter methylation of CpG islands via IL-1b can silence the *NF2* gene, which has pivotal roles in tumorigenesis and the development of WHO grade I meningiomas (35, 54). However, a recent study found the *NF2* promoter methylation in only one of 49 tumors examined, and only one of 40 examined CpG sites harbors the feature of this tumor, suggesting that *NF2* methylation did not play a major role in meningioma development (55). Similar to the *MEG3* gene, biallelic loss and promoter methylation has been observed in high-grade meningiomas, but only allelic loss correlated with gene silencing (53). Therefore, it is difficult to deny that both mechanisms are involved in this process, though it is necessary to know which one plays a more essential role. Some alterations of genes are affected by DNA methylation, while others are affected by different mechanisms.

### Other Aberrantly Methylated Genes

It is well known that epigenetic changes can affect both gene expression and the function of a protein product (56). To date, several studies have reported that the methylation of gene promoter CpG dinucleotides (CpG islands) have been connected to the WHO grades and prognosis in meningiomas. They have shown that the inactivation of transcription occurs when the promoter region of a tumor-related gene has been methylated, leading to the silencing of gene expression (53, 57, 58). Other studies have reported that global methylation might have an influence on tumor recurrence (22, 49). Generally, alterations of DNA methylation in meningiomas have two mechanisms: hypermethylation and hypomethylation. Hypomethylation, however, is much less common compared with hypermethylation (53). Each type of methylation has its own target genes that cause a change in meningioma aggression. Sometimes, these two mechanisms may co-exist in one sample. Therefore, additional studies are needed to figure out which mechanism is more important, even though both mechanisms have a function on gene mutation. In addition, some studies have shown that non-CpG island methylation plays an important function in gene expression (59, 60). However, further studies are warranted to establish the mechanism and function of non-CpG island methylation.

### The Possibility of Using These Genes as Predictors of Prognosis

Given results from previous studies, a large number of genes have been found to have aberrant methylation, some of which have influenced gene expression, resulting in tumorigenesis. Promoter methylation is the most common event in meningiomas and several genes are related to this pattern. Hence, in this section, we divide mechanisms into two groups (hypomethylation and hypermethylation) for further discussion (Table 5).

**TABLE 5:** Aberrant genes in meningiomas.

Gene	Altered DNA methylation	Role
uPA	Hypomethylation	Aggression
IGF2BP1, PDCD1	Hypermethylation	Increase malignant potential
HOXA7/HOXA9/HOXA10	Co-methylation	Progression and aggression
P73, RASSF1A, MAL2	Hypermethylation	Malignant transformation
p53, p14ARF, MEG3	Hypermethylation	Progression
CDKN2A, NDRG2, TIMP3	Hypermethylation	Progression and recurrence
THBS1	Hypermethylation	Angiogenesis
MGMT, WNK2	Hypermethylation	Unclear

#### Hypomethylation

##### Global methylation

To the best of our knowledge, global methylation has not been widely studied in meningiomas, and only a few groups have reported it (39, 49, 61). Among them, Gao et al. were the first to analyze whole-genome DNA methylation across three subtypes of meningiomas. After assessing DNA methylation in 19 primary brain tumor samples (10 benign, five atypical, and four malignant meningiomas), they found increased global DNA hypomethylation from grade I through III meningiomas, which was in line with gene expression results. These results were similar to Vengoechea et al. The latter concluded that high-grade meningioma harbored more global hypomethylation (49, 61, 62). Although global DNA hypomethylation can distinguish malignant meningiomas from atypical and benign ones, it cannot separate atypical and benign tumors. Thus, it may potentially serve as diagnostic biomarkers for only malignant tumors.

##### Aggression: urokinase plasminogen activator (uPA) and PAI-1

The uPA system plays an important role *in vivo*, such as in wound healing, embryogenesis and tumor progression, and metastasis (63). The expression of uPA has been linked to methylation of the uPA promoter in breast and prostate cancers (64, 65). PAI-1 is an inhibitor of uPA, and uPA/PAI-1 has been reported to contribute to glioma invasion and malignant progression (66, 67). Kandenwein et al. studied 65 tissue samples of meningiomas from 58 patients and found that the relationship between the expression of uPA and PAI-1 reached significance. Both protein expressions were significantly correlated with WHO grade. However, PAI-1 showed a highly significant correlation with prognosis, when setting 6 ng/ml as a cut-off of PAI-1 levels. The samples below this level were not recurrent in this study, demonstrating PAI-1 as a possible prognostic marker for meningiomas. Nevertheless, there was no correlation between the clinicopathological data and uPA promoter methylation (43). In fact, on the contrary, Arai et al. identified that uPA expression was inversely correlated with uPA promoter methylation levels (68).

According to results from some studies, the increased expression of uPA proteins has been shown to have a significant negative correlation with promoter methylation and positively correlated with WHO grade, malignant invasion, and recurrence (43, 69). Some studies even show a radiation-induced overexpression of uPA in meningioma cells, suggesting an additional level of regulation (70, 71). Velpula et al. (72) and Goetz et al. (73) pointed out that the expression of uPA has been related to radiation-induced hypomethylation. Therefore, patients with aberrant uPA gene methylation should carefully consider radiotherapy.

##### Hypermethylation

DNA hypermethylation means a specific site, that is unmethylated under normal conditions, has become methylated. And it always occurs in promoter CpGs islands. In meningiomas, several genes have an association with this epigenetic mechanism and show various influences on its progression and recurrence.

##### Increased malignant potential: IGF2 mRNA binding protein 1 (IGF2BP1), programmed cell death 1 (PDCD1/PD-1), NDRG2 and TIMP3

IGF2BP1, which belongs to the VICKZ family, is an RNA-binding protein that is implicated in tumorigenesis by influencing the translocation and stability of mRNA in some cancers (74). PDCD1 is a negative regulator of immune responses and likely plays an important role in the progression of many diseases (75), such as rheumatoid arthritis (76). Vengoechea et al. analyzed 49 samples from three grades of meningiomas and identified nine genes that exhibited the largest absolute difference in methylation intensity. Among them, the expression of IGF2BP1 and PDCD1 proteins were sharply decreased, indicating that both these genes were associated with the malignant potential of the tumor. These results suggest the potential ability of CDKN2A as a recurrence predictor. However, Aydemir et al. did not find any statistically significant relationship between hypermethylation of the *CDKN2A* gene and histopathologic subtype, WHO grade, and recurrence (77). Therefore, studies encompassing a larger series still need to evaluate whether or not CDKN2A alterations can be used as biomarkers of recurrence in meningioma.

Regarding *NDRG2*, previous research has indicated potential associations between this gene and the malignant progression of tumors. Several studies have documented that the loss of NDRG2 expression is significantly associated with hypermethylation of the NDRG2 promoter (78–82). Lusis et al. pointed out that hypermethylation of the NDRG2 promoter is described in a subset of lower-grade meningiomas, including clinically aggressive atypical meningiomas (78). Das et al. found that NDRG2 was marginally expressed or even undetectable in anaplastic meningiomas (83). Skiriute et al. demonstrated that the expression of the *NDRG2* gene was significantly reduced in primary and recurrent atypical/WHO grade II compared with primary benign/WHO grade I meningiomas (47). However, Majchrzak-Celiñska et al. questioned the reliability of NDRG2 as a diagnostic biomarker due to the fact that its methylation levels were only slightly elevated in comparison to the common brain tissue (84). Overall, these studies illustrate the heterogeneity of *NDRG2* methylation, and further studies are needed to determine the function of *NDRG2*.

*TIMP3* is located on 22q12.3 and codes for a protein that can specifically inhibit matrix metalloproteinases *(MMPs)* via covalent binding to the active site of the enzymes and reduces the invasiveness of tumor cells (85, 86). *MMPs* contain several classes of proteases and the expression of *MMPs* correlates with tumor stage, increased invasion, and metastasis (87). The allelic losses on 22q12 are associated with *TIMP3* hypermethylation and transcriptional downregulation. The promoter hypermethylation of *TIMP3* was associated with a more aggressive and higher-grade meningioma phenotype and poor prognosis (88, 89). There is a growing body of evidence indicating that *TIMP3* methylation could be an epigenetic marker of meningioma progression. Pham et al. analyzed 50 meningiomas (27 Grade I patients, 11 Grade II patients, and 12 Grade III patients) and found that hypermethylation of *TIMP3* varied between anaplastic (67%), atypical (22%), and benign (17%) meningiomas (90). Bello et al. reported that Grade I tumors had less aberrant methylation than Grade II or III meningiomas (33). In contrast, Liu et al. investigated the same chromosomal region but did not find any hypermethylation of *TIMP3* in meningiomas (35). Though there was some evidence to indicate that the methylation of *TIMP3* gene is associated with a shorter time to recurrence, Linsler et al. pointed out that there was no correlation of *TIMP3* hypermethylation with tumor recurrence or WHO grade (88). Due to the non-uniform approach needed to detect DNA methylation, several studies have observed different results and further research needs to be conducted in order to establish a standard definition for methylation to solve the contradictions in the *TIMP3* gene.

##### Angiogenesis: the thrombospondin 1 (THBS1)

The *THBS1* gene is thought to inhibit angiogenesis by disrupting the motility of endothelial cells and inducing their apoptosis (91). Transcriptional silencing of THBS1 has been shown to be related to promoter hypermethylation (92, 93). One study reported that the silencing of this gene via hypermethylation can promote angiogenesis in tumor cells (94). However, this remains controversial. Bello et al. found that 54% of Grade III meningiomas and 30% of intracranial meningiomas demonstrated hypermethylation of the *THBS1* gene. However, they did not find any association between hypermethylation and WHO grade (33). Liu et al. did not find any *THBS1* gene hypermethylation (33), and the true role of the *THBS1* gene in meningiomas remains a mystery.

##### Unclear function: WNK lysine deficient protein kinase 2 (WNK2) and O6-methylguanine–DNA methyltransferase (MGMT)

Many genes have been found to be methylated in meningiomas, but whether they correlate with tumor progression remains a mystery. This is particularly true for two genes: *WNK2* and *MGMT*.

WNK2 is a member of the WNK subfamily of protein kinases (95, 96), which negatively regulates EGF-induced activation of the ERK/MAPK-pathway and the downstream cell cycle progression (97). In recent years, studies have shown that *WNK2* is a specific tumor-suppressor gene for brain tumors and its downregulation is significantly correlated with the presence of promoter methylation (98). Jun et al. analyzed 22 meningioma samples, suggesting that WNK2 was aberrantly methylated in a large proportion of grade II and III meningiomas. With further study, they found that dense aberrant methylation was associated with decreased WNK2 expression in these meningiomas and that aberrant DNA methylation existed in approximately 60 CpGs in the 3′ part of the island, with very little methylation in the 5′ region. Therefore, aberrant methylation of the 3′ region may silence WNK2 expression (99). As in infiltrative gliomas, WNK2 has been identified to be silenced by promoter methylation in most samples (100). These studies show WNK2 as a candidate predictor of meningiomas but also put forward that other mechanisms, such as signal path interference, might affect the expression of WNK2.

Research has shown that promoter hypermethylation of the *MGMT* gene can be a predictor in glioblastoma multiform (GBM) (101). Recently, several studies have reported hypermethylation of MGMT in meningiomas. Liu et al. reported 6% MGMT promoter hypermethylation in a group of 48 meningioma cases (35). Aydemir et al. (77) and Bello et al. (33) showed that MGMT was methylated in 11.1 and 16%, respectively, of their samples, though they did not find any significant correlation between methylation and tumor grade. However, Robles et al. had an entirely different outcome. They showed that none of the samples harbored MGMT promoter methylation (102), similar to Jabini et al. (103).

There are many studies that suggest opposite opinions, and the functions of these two genes in meningiomas still need to be uncovered. There need to be subsequent studies to figure out the relationship and mechanisms between these genes and meningiomas.

### The Role of Methylation in Meningiomas

Studies have shown that molecular subsets of meningiomas could be identified by their epigenetics (22, 49), and epigenetics can uncover tumor progression. In this review, we discuss DNA methylation in meningiomas. From previous studies, we can conclude two major roles of methylation in meningiomas.

#### Prediction

The first role of methylation in meningiomas is the ability to predict tumor recurrence. Genes like *NDRG2* and *TIMP3* have been proven to be associated with recurrence (47, 88). Meningiomas with these methylated genes indicate a shorter time to recurrence. In order to prove the role of DNA methylation in meningiomas, Nassiri et al. used DNA methylation profiles of clinically annotated tumor samples among multiple institutions to develop a methylome model of 5-year recurrence-free survival (RFS). They also combined a methylome model with established prognostic clinical factors to obtain a 5-year meningioma recurrence score through a nomogram. They found that adding the methylome predictor enhanced the discriminatory ability of the nomogram (4). Additionally, a scoring system established on a scale of 5–15 points that was comprised of three stages depending on the methylation values of the five chosen genes [*HOXA6*, *HOXA9*, *PENK*, *UPK3A*, and *IGF2BP1* (39)] also verified the value of methylation in meningiomas. The samples in their study that had scored lower than 9 points demonstrated significant differences in the PFS curve compared to samples that scored more than nine points (22). Due to the non-uniform cognition of aberrantly methylated genes, studies up to now have been independent of each other, but both types of studies have uncovered the potential of DNA methylation to predict the recurrence of meningiomas.

#### Risk Stratification

The secondary role of DNA methylation is to stratify meningiomas. It is hard to classify meningiomas into three groups like benign, middle, and malignant using only histological grading. The new methylation-based classification has demonstrated the ability to divide tumors into different risk groups (6). Genes like *MAL2* and *RASSF1A* have been connected to malignant transformation (34, 49, 84) and the benign tumors harboring these genes have the potential ability to become malignant. Therefore, these tumors should be taken seriously. However, using methylation profiling alone to assess the tumor was not precise because other factors should be taken into consideration as well, such as Ki-67 and clinical characters. Moreover, with a deepening of research, the importance of methylation to stratify high-risk patients gradually emerged. For example, the presence of three or more hypermethylated TSGs has been shown to be a useful biomarker for risk stratification in meningiomas (104). Hence, utilizing methylation profiling to identify high-risk patients is possible, but more integrated and supportive studies are needed.

## Hypothesis: Methylation Profiles Could Be Used in Addition to the Who Grade and Simpson Grade to Identify Tumor Recurrence More Accurately?

Nowadays, clinicians often judge the characteristics of meningiomas by histological grade alone. While most patients choose to undergo a pathological examination, some patients may have gene detection after the operation. According to NCCN clinical guidelines, subgroups of patients should receive radiotherapy after the operation, such as those with a WHO grade III tumor and those who have undergone partial excision of a WHO grade II tumor. Some patients who detect the abnormal genes could accept molecular targeting treatment, while other therapies, such as chemotherapy and hormonotherapy, depend on the situation. The follow-up plan depends on the WHO grade, Simpson grade, and personal situations, but all these criteria are very general and sometimes the decision depends largely on the clinicians’ experience. Although the new methylation-based classification may take a long time to be applied in regular clinical practice, it may improve treatment decisions as the methylation-based classification is more accurate than WHO grading (6). Our review has highlighted certain genes that are associated with the progression and prognosis of meningiomas through DNA methylation. Gene expression is controlled via hypermethylation or hypomethylation which, in turn, affects tumor evolution. Based on previous studies, methylation signatures have been proven to be a predictor of meningioma prognosis and the use of methylation signatures to stratify meningiomas has yielded better results than using WHO grade or Simpson grade (6). Hence, methylation profiles could be used in addition to WHO grade and Simpson grade to identify tumor recurrence more accurately.

**FIGURE 1:**
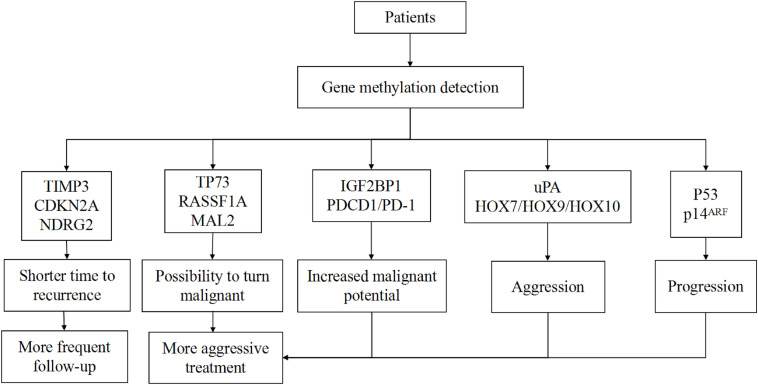
Patient classification based on genes.

If we combine the aforementioned scoring system with the WHO and Simpson systems, using *HOXA6*, *HOXA9*, *PENK*, *UPK3A*, and *IGF2BP1* genes to distinguish patients with a high probability for recurrence, these patients may receive more aggressive treatment and more frequent follow-up plans, and some recurrence can be detected earlier than before. However, this scoring system has certain drawbacks in that the chosen five genes are not typical for all grades of tumors. It is also hard to detect the global gene methylation profile for every patient due to economic capacity and technical conditions. Currently, there are several techniques for global DNA methylation profiling, such as WGBS and RRBS. For now, WGBS is the gold standard method to investigate every CpG site in the genome (105). However, an analysis showed more than 70% of sequenced reads did not give useful information (106). For one sample, WGBS needs two lanes of sequencing using the Illumina HiSeq system to obtain a tenfold average coverage of CpG sites, which costs ∼US$6000 (107). Due to the substantial cost and large volume of raw data, applying WGBS in clinical practice is not possible in the short term. Other methods such as RRBS are cost-effective, but the region is limited to the enzyme recognition sites (108). Though new technologies are constantly being invented, these are not the best methods for clinical application. Therefore, it is necessary to establish a reliable system that contains the chosen aberrantly methylated genes that are associated with the invasiveness of meningiomas. It is important to divide these genes into several groups according to their function on the progression of meningiomas; benign tumors which harbor *TP73*/*RASSF1A* hypermethylation, for example, are more likely to turn malignant while the hypermethylation of *TIMP3* marks a shorter time to recurrence. We hypothesize that putting the same functional genes together and choosing landmark genes for detection can help create a special report for each patient with regards to tumor aggressiveness and risk of recurrence. This information can help clinicians set an optimal therapeutic regimen and follow-up plan for each patient (Figure 1).

With further studies, an increasing number of genes are found to be methylated during the meningioma progression. Additionally, controversial genes such as *THBS1* and *MGMT* can be added to this system to help clinicians judge the situation of each tumor. Though the correlation between methylated genes and histological subtypes has not yet been found, the relationship between the WHO grade and DNA methylation class has been explored in several studies (6). Other relationships, such as the relevance between specific histological subtype and methylation class, have been studied as well (50). Further studies should continue to focus on this hotspot. Therefore, the system can be improved to accurately correspond to each subtype and specific abnormal genes can be detected in limited conditions. It is more efficient for doctors to describe the features of tumors using methylation profiles, genetic mutations, or histological subtypes. As it takes a long time to establish criteria based on methylation, the emphasis now should be on forming a system that contains useful genes that can improve the existing grading. There needs to be a uniform standard to define methylation and a large number of samples of whole grade tumors. It may take a long time, but it can be established step-by-step.

## Conclusion and Perspectives

Several genes have come to be considered as involved in the progression of meningiomas through methylation. With the results from the detection of gene methylation, an increasing amount of new genes are connected to tumorigenesis. During this research, it has been shown that different genes have different functions. While some genes already have a clear role, other genes (i.e., TIMP3, THBS1, and MGMT) remain controversial and need more research. The incorporation of several genes has been studied successfully to predict survival times and recurrence risk in meningioma patients. A new classification system focused on DNA methylation is able to identify meningiomas more accurately. Though there are lots of grading systems, it seems they are independent of each other and it is difficult to integrate them or make a comprehensive standard for the identification of various meningiomas. Thus, there needs to be a revised system that can improve present grading. Based on conclusions from other studies, we believe that combining a new system that contains several remarkable methylated genes with current grade systems (WHO grade and Simpson grade) can help clinicians choose an individualized therapeutic regimen (like surgery, radiotherapy, or chemotherapy) and follow-up plan for each patient. We have reviewed several grading systems and a series of methylated genes in meningiomas and concluded the ability of methylation profiling to identify various meningiomas. This leads us to put forward a hypothesis that methylation profiling can serve as a supplement to clinical predictors and provide a more accurate prediction of recurrence risk.

## Data Availability Statement

The original contributions presented in the study are included in the article/supplementary material, further inquiries can be directed to the corresponding author/s.

## Author Contributions

AS, XW, and LC supervised the research, led the discussion, and wrote and revised the manuscript. All authors participated in analyzing and discussing the literature, commenting on, and read and approved the final manuscript.

## Conflict of Interest

The authors declare that the research was conducted in the absence of any commercial or financial relationships that could be construed as a potential conflict of interest.
